# Chemometric Assessment of Soil Pollution and Pollution Source Apportionment for an Industrially Impacted Region around a Non-Ferrous Metal Smelter in Bulgaria

**DOI:** 10.3390/molecules24050883

**Published:** 2019-03-02

**Authors:** Dimitar S. Dimitrov, Miroslava A. Nedyalkova, Borjana V. Donkova, Vasil D. Simeonov

**Affiliations:** Faculty of Chemistry and Pharmacy, University of Sofia, 1 James Bourchier Blvd., Sofia 1164, Bulgaria; d_100@abv.bg (D.S.D.); mici345@yahoo.com (M.A.N.); bordonkova@abv.bg (B.V.D.)

**Keywords:** trace metals, transfer factor, multivariate statistics, pollution source identification, biomonitoring

## Abstract

The present study deals with the assessment of pollution caused by a large industrial facility using multivariate statistical methods. The primary goal is to classify specific pollution sources and to apportion their involvement in the formation of the total concentration of the chemical parameters being monitored. This aim is accomplished by intelligent data analysis based on cluster analysis, principal component analysis and principal component regression analysis. Five latent factors are found to explain over 80% of the total variance of the system being conditionally named “organic”, “non-ferrous smelter”, “acidic”, “secondary anthropogenic contribution” and “natural” factor. The apportionment models designate the contribution of the identified sources quantitatively and help in the interpretation of risk assessment and management actions. Since the study takes into account pollution uptake from soil to a cabbage plant, the data interpretation could help in introducing biomonitoring aspects of the assessment. The chemometric expertise helps in revealing hidden relationships between the objects and the variables involved to achieve a better understanding of specific pollution events in the soil of a severely industrially impacted region.

## 1. Introduction

The industrial impact of non-ferrous metals mining and smelting activities on heavy metal pollution and its long-term effect on the environment and population is the object of a significant number of studies [1,2,3,4,5,6,7,8,9,10,11,12]. These investigations show that the contamination emitted to the environment affects not only the neighboring locations but a much larger region that is dependent on geographical specification (landscape, height above the sea level, underground waters, river catchments), transportation net, climate indicators (wind direction, temperature, humidity) as well as the size, chemical and mineralogical composition emitted by the smelters’ particulate matter. The seriousness of the problem is supported by evidence for high concentrations of heavy and toxic metals in soils in the areas around historic smelters even if they have not been operational for centuries [12]. Because heavy metals are also a natural presence, any assessment of their industrial impact should be keep in mind in the background of geology as well [2,4,12].

The uptake of contaminants in the plant species via roots or by direct atmospheric deposition onto the plant surfaces are two of the major exposure routes for humans, along with ingestion/inhalation of the aerosols and dust re-suspension. [4,6,7,8,9,11]. Studies indicate that the uptake and bioaccumulation of metals depend not only on the plant type but also on many other factors such as soil composition and physicochemical properties, climate, anthropogenic sources like road traffic, and transport of long-distance aerosols.

An important issue is source tracing in the smelter-affected environmental systems to apportion the pollution impact of the smelter from other sources. It helps in decision making and risk management over the whole region of interest. One of the methods for tracing the origin and fate of contaminants near non-ferrous metal smelters is the Isotope Ratio Analysis, which is well cited and discussed in [12]. The most used Pb isotope systems are referenced in the following studies [2,11,13,14,15]. However, this approach has some limitations [12,15] that require additional studies of local mineralogical composition.

Chemometric methods for data mining and modeling, such as principal components analysis (PCA) or principal components regression (PCR) are often used for the identification of factors influencing the soil pollution and the bioavailability of heavy metals [9,10,11,16]. Some useful chemometric methods for source apportionment include [11,17,18].

The present research studied the soil and cabbage leaves heavy metal pollution in the area around a large-scale non-ferrous metal smelter (the largest Bulgarian producer of lead and zinc, the KCM AD). The smelter has been in operation since 1961. Although significant technological improvements were introduced in the early 1990s, in order to reduce smelter emissions, numerous studies around 2004 indicate that the local soil still possesses high accumulated heavy metals pollution (especially Cd, Pb, Zn) due to the long-term operation of the smelter, which is reflected in human health and life quality [1,2,19,20]. Based on the Pb isotopic analysis, the authors of [2] concluded that the primary source of atmospheric lead deposition is the ore material used in the smelter and up to 12% could originate from other sources such as petrol lead. To assess the alternations throughout the years and the impact of the smelter, the present research aims to determine the soil and cabbage leaves heavy metal pollution in the area around the smelter using chemometrics. The soil and cabbage (*Brassica oleracea*) samples were taken over three consecutive years from several places in different geographical directions in the cross point around the smelter. Soil structure and physicochemical parameters (soil capacity, organic and total organic carbon, pH, CEC and others), soil nutritional components (Mg, P, K, N), soil and leaves pollution by Hg, Cd, Zn, Cu, Pb were monitored. The chemometric expertise applied had the following goals: 1) to find patterns of similarity between the soil parameters, nutritional components, soil, and leaves contamination by heavy metals, as well as between locations using clustering approaches; 2) to identify the potential sources of heavy metals applying principal component analysis and the source apportioning model. Thus, it becomes possible to quantify the actual contribution of the non-ferrous metal smelter to the overall pollution of the studied area since the apportionment procedure allows us to identify other potential sources of pollution, keeping in mind not only the industrial activity but also agricultural impacts, traffic routes, high population density, long-distance transfer, etc.

The specific novelty in the present study is the introduction of apportionment procedure (being typical for air pollution receptor modeling) that separates the overall soil pollution into ‘fractions” originating from different anthropogenic (or natural) sources in the vicinity of the receptor region.

## 2. Input Data Set

### 2.1. Objects

**Figure 1:**
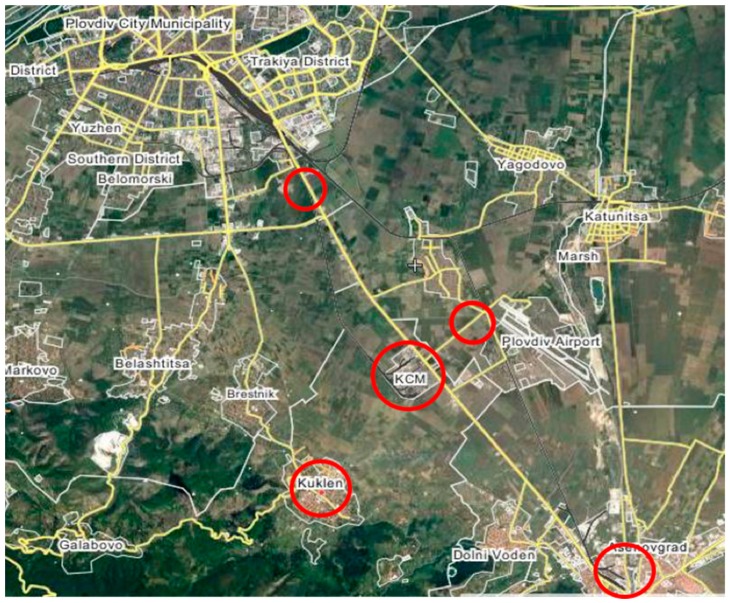
Sampling locations.

The non-ferrous metal smelter (elevation 179 m) is located at the в south-western part of the Thracian Plain nearby the road connecting the second largest town in Bulgaria, Plovdiv (180 m), and a smaller town called Asenovgrad (231 m) (Figure 1).

In the vicinity of the smelter is the agricultural land of the village of Kuklen, which is supposed to be highly contaminated. This location is noted as KAGRI. For comparison, the other samples (location K) were taken from the soil in the village Kuklen (287 m), situated about 4 km south of the smelter, on the outskirts of the northern slopes of the Rhodope Mountains and upwind of the smelter. The next location, KRM, is in the plain (Krumovo, 180 m) in the north direction from the smelter. To study the impact of the transport nearby the smelter, as well as the effect of the other human activities the other two locations that were chosen (Plovdiv and Asenovgrad) lay on the road and were the same distance (7 km) from the smelter but in opposite directions: NW and SE. The agriculture land in the vicinity of Plovdiv is marked as PB. The land from Asenovgrad is noted as AS. Spot samples from soil and leaves were taken from the urban center of Plovdiv (location PBUR—only one sample).

The samples were collected over a period of three consecutive years (2010–2012), and they are marked, for example, as KAGRI_10. The first is the location, and the number shows the year of sampling. The total number of objects is 16. The input data set is presented as Table S1 in the Supplementary part. The basic stastistics table is also given there as Table S2.

### 2.2. Cheimical Variables

Topsoil samples and samples of cabbage were collected in the period of September 2010–September 2012. The 12 soil parameters, as well as the content of six heavy metals in the soil and cabbage leaves, are monitored. All they form 22 variables in the input data set, conditionally combined into four groups, shown in Table 1.

## 3. Results and Discussion

The original data (including calculated transfer factors) are available on request. The transfer factor was not included in the chemometric expertise since it was calculated from the concentration values of the chemical variables in the soil and the plant leaves and, in this way, was correlated to these variables. It is used for some explanation of the uptake.

### 3.1. Cluster Analysis

Hierarchical and non-hierarchical cluster analyses treated a data matrix consisting of 16 objects and 22 variables. Both clustering approaches give four significant clusters for the variables and objectives with the same members of each identified cluster. This proves that the separation into four groups of similarity is stable and reliable. The clustering of the 22 variables will be shown as an example for hierarchical clustering, and grouping of the objects (locations and year) will be presented as an example for the non-hierarchical mode.

#### 3.1.1. Clustering of the Variables

**Figure 2:**
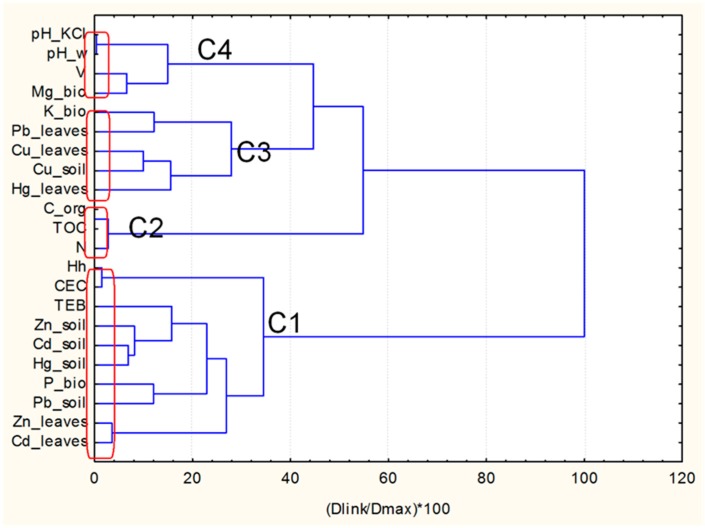
Hierarchical dendrogram for four clusters of variables. The conditional name of the clusters are (bottom to up): C1—structural and soil pollution factor; C2—organic factor; C3—cabbage leaves Pb, Cu, Hg accumulation factor; C4—soil acidity factor.

In Figure 2 the graphical output of the hierarchical clustering mode (dendrogram) is disclosed.

It might be concluded that four significant factors regulate the data structure. Depending on the prevailing type of variables, the included elements could be conditionally named as follows: structural and soil pollution factor (C1), organic factor (C2), cabbage leaves Pb, Cu, Hg accumulation factor (C3), and soil acidity factor (C4).

The soil pollution (C1) with heavy metals is linked to the soil structural characteristics, which is consistent with the results from the Pearson’s correlation analysis [6]. The variable “leaves” for Zn and Cd belong to the same cluster, showing that these metals are accumulated in the cabbage leaves by the high level of correlation. Copper “soil” and “leaves” variables are also linked together but, in another cluster, (C3). For Hg and Pb one finds the separation between these two types of variables. It should be noted that the same relationship between Cd, Zn, and Cu content in 44 soils and the majority of the 283 vegetables was established in [9]. The trend in the calculated soil-to-plant transfer factor decreases in the order Zn > Cd > Cu > Hg > Pb, being much higher for the first two metals than for the Hg and Pb, and middle for Cu. Such a ratio between accumulation factors for Zn and Cd to that for Hg and Pb is established in Refs. [7,11]. The separation of “soil” and “leaves” variables for Hg and Pb is logical, taking into account their low transfer factor and reported conclusions, that the most important source for vegetable pollution with these metals is the airborne metal-containing dust from the smelter and distant aerosols [1,6,7,9,11,21].

The nutritional components (Figure 2) are not parted as a formation in a single cluster but are dispersed in along different groups. The incorporation of these components in the input data set allows us to reveal a specific pattern of similarity: the leaves pollution with a given heavy metal is connected to the given soil nutritional component. The accumulation of Zn and Cd (cluster 1) is linked to the phosphorus nutrient P_bio, while leaves pollution with Cu, Hg and Pb (cluster 3) are related to nutrient K_bio. The transfer pathway for Zn and Cd from soil to edible tissues of vegetables is through root uptake, and the phosphorus has an essential significance in the early period of plant development by the root growth. The dominant pathway for entering of Pb and Hg in the vegetables is the uptake of deposited particulate matter through the above-ground plant tissues. Therefore, the leaves pollution depends on the K nutrient, which is vital for photosynthesis and is involved in osmoregulation.

**Figure 3:**
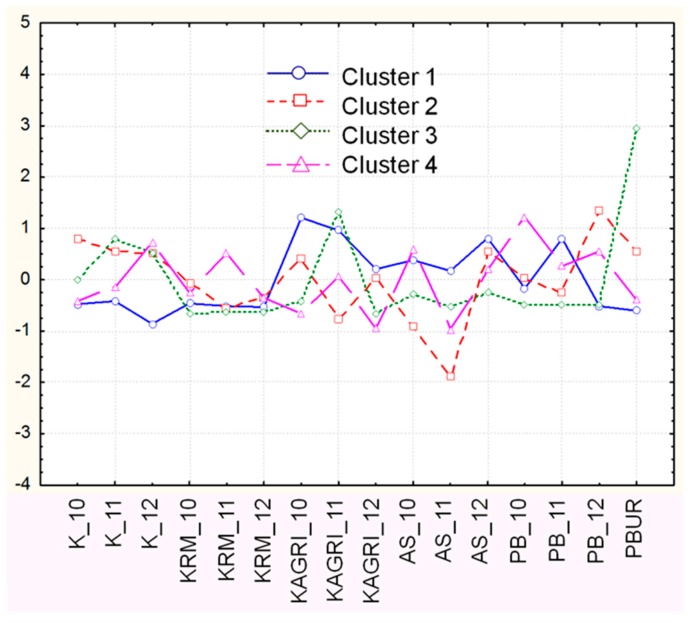
Averages (normalized) of each cluster of variables for each sampling location.

To indicate the reason for the data structure observed, the averages of z-standardized values of the variables for each cluster and each sampling location are presented in Figure 3. It should be noted that this presentation is obtained by K-means non-hierarchical clustering based on the preliminary hypothesis for the existence of 4 clusters of variables (hierarchical method). Indeed, the non-hierarchical mode for the preselected four groups of variables gave the same results as the hierarchical approach.

One could easily find the relationship between the different factors and the sampling locations and conditions. For instance, cluster 1 (structural and soil pollution factor) shows a positive deviation in respect to the limited zero value for all samples in the vicinity of the non-ferrous metal smelter (KAGRI) and from the town Asenovgard (AS) for the period studied. It should be noted that the soil in the Asenovgrad area is clay-rich and naturally enriched with Pb and probably other heavy metals [2]. Our data for metal contents in the soil prove this because in many of the AS soil samples the specific metal content (Zn, Cu, Pb, Hg) is higher than or equal to that around the smelter. However, the highest deviations for this cluster are registered for locations such as KAGRI, showing the industrial impact of the lead-zinc smelter. The locations K and KRM show negative differences from the conditional zero value.

The trend in the deviations of soil carbon and N nutrient (C2) is opposite to that of the soil pollution; logically, taking into account their origin and their chemical and biological specificity.

The trend in the z-standardized values for cabbage leaves Pb, Cu, Hg accumulation factor (C3) on first glance is incomprehensible, because in almost all cases the deviations from the average values are opposite to that of cluster 1. However, it can be explained with the low transfer soil-to-leaves factor for Hg and Pb and their entrance pathway mentioned above. Other anthropogenic activities mainly traffic, and roadside pollution affects the levels of leaves pollution. The evidence is the maximum positive deviation established for the Plovdiv urban area and high standards for location Kuklen. As has been shown in [2] the Cd, Zn, Cu, Pb contents in roadside soil beside position Kuklen were higher than in the surface soil level (0–20) in period 2003–2004, as a result of ore transport from the mine to the smelter.

The acidity factor (C4) is distributed quite homogeneously in the area studied because the acidity of almost all the soil samples is the same, which is close to neutral.

#### 3.1.2. Clustering of the Objects

The classification of the objects (performed by the non-hierarchical mode) from the sampling net follows spatial patterns: one detects the grouping of the locations of Krumovo and agricultural land of Plovdiv; agricultural land of Kuklen as subject to industrial pollution (KAGRI) but separated from the property of the village Kuklen (K). The fourth cluster includes samples from the vicinity of the town of Asenovgrad.

The clusters are as follows:

C1 *(KRM_10, KRM_11, KRM_12, PB_10, PB_11, PB_12)*
C2 *(KAGRI_10, KAGRI_11, KAGRI_12, AS_10)*
C3 (*K_10, K_11, K_12, PBUR*)
C4 (*AS_11, AS_12*)

**Figure 4:**
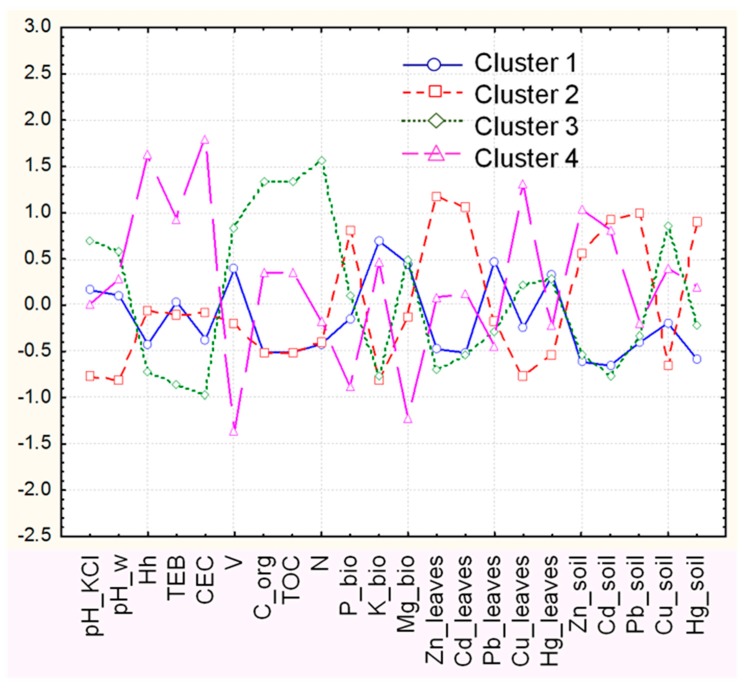
Averages (normalized) for each variable and for each cluster of locations.

It is important to determine the discriminant variables responsible for the object clustering. In Figure 4 the mean values (z-standardized) of the measured variables included in each of the identified clusters of location are presented.

It is shown that for locations KRM and PB (C1) almost all variables are distributed uniformly around the mean value. Specific features are the lowest level of soil pollution and the lowest level of the organic factor. One and the same level of organic element is seen for the all the samples of KAGRI-area in the vicinity of the smelter (C2), however the discriminating variable for this cluster is the highest positive deviations for soil pollution, with almost all studied heavy metals: Zn, Cd, Hg, Pb. Surprisingly, copper has the lowest negative deviation. The soils in cluster 3 (locations K and PBUR) could be considered as “uncontaminated,” except at a high level of Cu. These locations are distinguished by the highest levels of soil organic factor, which shows the importance of the geographical area and the disposal of domestic waste. Particular structural soil characteristics allow the separation of samples from location AS (C4). As mentioned above, in the vicinity of the town of Asenovgrad the soils are more clay-rich than at the other sampling locations, and the ability of soil to adsorb cations is greater. Besides, as reported in [2] the soils near Asenovgard have high baseline geogenic concentration of Pb and are more polluted with Zn, Cd, and Pb than that in Kuklen. Our chemometric analysis supports the above statements—the positive deviation for soil pollution correlates with the highest variation in soil structural parameters.

It should be noted that Asenovgrad is located in the River Chaia valley originating from the Rhodopa Mountain. The river catchment is fed by the underground waters from a metal-rich region (with massive Pb-Zn ore deposits and Ag, Au, Cu mineralization). Highest averages for zinc and lead for the period studied are registered for location AS. So, the soils nearby the river, as well as the agricultural lands that are irrigated by the river и by local wells are expected to be enriched with heavy metals.

The chemometric approach reveals clearly that the accumulation of the heavy metals in the cabbage leaves is related not so directly to the soil pollution but the dominating mode of uptake—through the roots or the aerial part of the plant. The deviation of Zn and Cd “leaves” variables is highest for most polluted with these metals’ locations KAGRI and AS (C2 and C4) and is lowest for “non polluted” by these metals location K, KRM, PB and PBUR (C1 and C3). The highest positive deviation for Pb and Hg “leaves” is observed for locations KRM and PB (C1), proving the role of the relief, wind direction and roadside pollution/traffic/for contamination of the cabbage with these metals. The role of the P and K nutrients for accumulations of the heavy metals commented on above can also be observed in Figure 4.

Particular attention ought to be paid to the deviations concerning the average value of the variable Cu_soil. For all location clusters, they are opposed to that of other heavy metals “soil” variables, indicating that the copper source in soil is different. Moreover, a particular pattern of similarity could be observed between the organic factor and Cu_soil for all of the above mentioned clusters. The highest positive deviation in the organic factor is connected with the highest positive deviation for the Cu_soil (cluster 3), while the lowest deviations for the organic factor (clusters 1 and 2) correlate to the lowest values for Cu_soil. This confirms the fact that Cu bonds strongly to the organic matter (OM) [22]. Besides, the deviations in the variable Cu_leaves are changed in the same trend, except for locations in cluster 4 (AS), where the level is higher than cluster 3 (K and PBUR). As discussed in [10] the organic matter content (more correct the “active OM”) may enhance heavy metal availability to the plants by increasing CEC in the soils, providing metal chelates. As we see, the highest level for all soil structural characteristics (CEC, TEB, Hh) is observed for cluster 4, so it could be assumed that the active OM for locations in this cluster is higher than passive OM (humus).

### 3.2. Principal Components Analysis

To identify the latent factors responsible for the data structure and the appropriate interpretation of the pollution sources in the region of interest, principal components analysis (Varimax rotation mode, normalized input data) was carried out. Since the primary goal is to interpret soil pollution impacts, the variables chosen for the chemometric analysis, in this case, were 13 for all sampling locations in consideration. Among the soil properties controlling the speciation and mobility of the heavy metals, the pH and organic matter content are considered very important [9,10]. Based on the results from clustering analysis (Figure 2) that suggest accumulation in the leaves is connected with the nutritional components, the latter were also included in the PCA. For convenience, soil structural parameters (Hh, CEC, and TEB) were omitted since they are not subjects strongly affected by anthropogenic impacts. Metal concentrations in cabbage leaves were also eliminated as it was shown that they strongly correlate to the pollution in soil.

In Table 2 the factor loadings for five principal components are presented. They explain nearly 90% of the total variance of the system. Factor loadings biplots are additionally presented as Figure S1 in the Supplementary part.

PC1 indicates statistically significant factor loadings for N, C_org and TOC (variance explanation 23.7%) and could be conditionally named the “organic” factor. It reflects the impact of the organic soil content on the pollution events and transfer processes.

PC2 shows the significant positive correlation between Zn, Cd, Hg, and to a lesser extent Pb. This is a convincing indication for the role of these elements in soil pollution by smelter operating (explained variance 20.2%). Obviously, the non-ferrous metal smelter is the source for this type of pollution in the region and, therefore, this latent factor could be conditionally named the “non-ferrous smelter” factor.

PC3 relates the pH condition of the soil both in water and KCl extracts. Non-significant correlation with Mg is observed. It explains 17.9% of the total variance and could be conditionally named the “acidic” factor. The soil acidity is almost constant and therefore no specific correlation with the metal pollution is found.

PC4 is of a more complex structure since it shows significant correlation between the soil nutrients P and Mg and to lesser extent with Pb and Hg (explained variance 15.5%). A simple conditional name could be “secondary anthropogenic contribution” factor, keeping in mind the role of fertilizers and pesticides, municipal wastes, local traffic pollution, distance aerosols and of course, sources related to ore transport. The coal combustion used for power plants and residential heating are main sources of anthropogenic Hg emissions in Bulgaria [23].

The last PC5 explains the other 12.1% of the total variance revealing a not very specific and straight forward relation between copper soil content and potassium. It could be assumed that this latent factor is responsible for the geogenic conditions (the Rhodope Mountain is a metal-rich ore of various combination of Mo, W, Fe, Pb, Zn, Ag, Au, Cu mineralization with the industrial importance of Pb-Zn ores) and burning processes (wood, forest fires, plant oils) in the region of interest. The conditional name suggested for this factor is “natural” factor.

### 3.3. Source Apportionment

In the next step of the chemometric expertise a source apportioning procedure (Thurson and Spengler APCS method [24]) was performed in order to assess the contribution of each identified source to the total concentration of each parameter. The results are presented in Table 3.

Each row of the table indicates the regression model obtained for each parameter involved. The model is of the type:

Y = a_0_ + Σa_i_PC_i_

where Y is the total concentration of a certain chemical parameter; a_0_ is the intercept; a_i_ is the regression coefficient showing the contribution of each identified source to the total concentration Y; PC_i_ is each identified source (as absolute principal components score).

The contributions of each source are given in % for easier interpretation. In the last column of the table the value of the multiple correlation coefficient R^2^ is given, which shows the percentage of explanation of the total concentration by the model (model validity). The intercept is a measure for the non-explained concentration of the identified sources (there are other sources in the region contributing to the total concentration or pollution impact).

The results from the APCS model suggested that the airborne metal-containing dust from the non-ferrous metal smelter operating leads to heavy pollution of Zn, Cd, and Hg in soils in the area studied. The cinnabar (HgS) deposits are concentrated in two regional stripes in the Rhodopa Mountain, one of which is the region where the ore source for the smelter is found [25].

As we see, however, the main source of Cu in the soils is natural, and this fact explains the observed strange deviation in the Cu_soil variable in respect to the other heavy metals, commented on in Figure 3. This is proved from the experimental data, showing the highest average Cu content in the soils from the village of Kuklen and the lowest average value for the location of KAGRI-smelter area. The natural source of Cu in the lead-zinc smelting areas in China is assessed in [6]. The natural source contributes to the other heavy metals except Hg concentration. A logical reason is that the Rhodopa Mountain is a metal ores-rich region with a highly developed mining industry. It is an area of the most considerable deposition of non-ferrous metal ores on the Balkans and represents one of the most significant manifestations of vein type Pb-Zn mineralization in the world. The underground waters, the springs, and the Chaia River (flowing through the town of Assenovgrad) are directed downhill, so, the soils as well as the agricultural lands that are irrigated from the river and wells are expected to be enriched with heavy metals.

The agriculture activity, industrialization, and urbanization are main sources not only for P and Mg but as the results show, for Pb pollution in the area studied. In the previous research on the contamination near the smelters, based on the Pb isotopic analysis, the authors [2] concluded that the primary source of atmospheric lead deposition in the grass in Kuklen and manure in Boyantsi (near Asenovgrad) is the ore used in the smelter-(up to 88%). The rest, approximately 12%, could originate from other sources such as petrol lead. Applying the APCS model for quantifying the impact of the smelter on the lead pollution of soils, we can distinguish the sources affecting it: 21.1% from the smelter, 61.7% from other sources (including industrial, traffic pollution, and ore transport, as well as long distance aerosols), 10.2% from natural origin, and 3.2% related to acidity.

The contribution of the acidic source to the soil pollution of Pb and Cu could be explained by the fact that soil leaching is dependent on soil pH, whereas the leaching of Cd and Zn in a greater extent is irrespective of soil acidity [12]. The lowest Cu content is registered for AS_11, when soil pH is acidic (4.25) while in all other years it is 6.35, and the Cu content is 45–47 times lower as compared to the different sampling years.

### 3.4. Comparison of the Metal Levels of Pollution in the Soil in the Vicinity of the Nonferrous Smelter for the Period 2000–2012

The soil pollution by toxic metals data from the beginning of the millennium [2] is compared in Table 4 with the average values of metal concentrations in the soils, collected in the period 2010–2012. Even though the smelter continues to be the primary source of Zn, Cd, Hg and to a lesser extent the Pb pollution, a comparison to previous data shows a significant decrease of the emissions of Zn, Cd, and Pb. Although our results do not relate the smelter production to copper pollution, it is readily seen that the copper content in the soil is significantly reduced. One of the possible reasons is the closure of the mine in the village of Zvezdel and the exchange of the ore source with another one.

## 4. Materials and Methods

### 4.1. Sample Collection, Preparation and Chemical Analysis

Topsoil (0–20 cm) samples (each sampling location indicated in Part 2.1. Objects) together with the samples of cabbage leaves (*Brassica oleracea*) growing on these soils were collected during the period September of 2010–September 2012. The 12 soil parameters, as well as the content of six heavy metals in the soil and cabbage leaves are monitored (see Table 1).

The soil pH was measured in a 1:5 suspension of soil in pure water and KCl suspensions (ISO 10390:2005) [26]. Equivalent calcium carbonate (%) and total organic matter content (TOM %) were determined according to ISO 10693:1995 and ISO 10694:1995, respectively [27,28].

The digestion of soil samples was performed by the adopted method of ISO 11466:1995 [29]. Three portions were weighed for each sample, and procedural blank was run during the procedure. The aqua regia soluble content of all the analytes was measured by Inductively Coupled Plasma Optical Emission Spectrometry (ICP-OES) (Perkin Elmer ICP-OES 6000, Waltham, MA, United States). For accuracy checking, two certified reference material Stream Sediments, STSD-1 and STSD-3, were digested in parallel. The obtained values for analytical recovery varied between 95% and 112%, which was considered as satisfactory.

The aerial part of the cabbage leaves was gently washed with Milli-Q water for approximately 3 min to remove soil and dust particles adhered to the plants. After washing, cabbage samples were air-dried at room temperature for 15 days and then thoroughly ground, milled, mixed, and uninformed to obtain representative samples. Plant samples were digested with 65% HNO_3_ and 30% H_2_O_2_ (USGS Test Method B-9001-95 [30]). The content of the elements was determined by ICP-OES and by Electrothermal Atomic Absorption Spectroscopy (ETAAS, Perkin Elmer, Waltham, MA, United States). The standard reference material ERM-CD 281 RYE GRASS was used to check the reliability of the results. The measured concentrations were in excellent agreement with the certified values (recoveries between 93% and 105%).

All reagents used were of analytical-reagent grade (p.a. Merck, Darmstadt, Germany). Milli-Q water (Millipore, Bedford, MA, USA) was used throughout.

### 4.2. Chemometric Methods

#### 4.2.1. Cluster Analysis

Cluster analysis (CA) is a well documented chemometric approach (both as hierarchical and non-hierarchical clustering) that reveals groups of similarity between a set of objects (samples) described by a certain number of variables (parameters, indicators) [31]. The hierarchical cluster analysis is a typical non-supervised pattern recognition technique that leads to spontaneous formation of clusters based on several preliminary steps such as standardization of the raw input data by a z-transform method (aiming elimination of the variable dimension on the process of grouping); determination of the similarity (distances) between the objects, e.g., by squared Euclidean distance; linkage of the objects into clusters, e.g., by Ward’s method of linkage; graphical output of the clustering as a plot known as the hierarchical dendrogram and, finally, the determination of the cluster significance, e.g., by the index of Sneath (1/3 or 2/3 Dlink/Dmax, where Dlink is a given distance of similarity and Dmax is the maximal distance in the set analyzed). In this way both objects or variables could be clustered and interpreted.

The non-hierarchical cluster analysis (very often a K-means approach is applied) is a supervised pattern recognition method whose aim is to collect the objects into a preliminarily determined number of clusters. The choice is usually the result of a specific hypothesis or expert opinion. According to the classical definition, a K-means cluster analysis has the goal to divide the set of n objects into predefined k number of clusters so that each object belongs to a group (cluster) with the nearest mean being the prototype of a cluster. After several iterations, each member of the set could be attributed to the preliminarily defined clusters. Again, Euclidean distance is used as a metric. There are some limitations to the application of K-means cluster analysis, especially when the preliminarily determined number of clusters is not well defined (by other methods or by scientific assumptions).

#### 4.2.2. Principal Components Analysis

Principal components analysis (PCA) is a typical projection method. It enables the reduction of the dimensionality of the space of the variables in the direction of the highest variance of the system, new variables being linear combinations of the previous variables, replacing the old coordinates of the factor space. The new coordinates are called latent factors or principal components. The interpretation of the new factors is the primary goal since they deliver useful information about hidden relationships within the data set. The results are indicated by two outputs, factor scores giving the new coordinates of the factor space with the location of the objects and factor loadings informing on the relationship between the variables. Only statistically significant loadings (>0.70) are essential for the modeling procedure.

The new principal components (latent factors) explain a substantial part of the total variance of the system for an adequate statistical model. Usually, the first principal component (PC1) explains the maximal part of the system variation and each additional PC has a respective contribution to the variance explanation but with less significance.

A reliable model usually requires many such PCs, so that over 75% of the total variation can be explained. In case of presented modeling, the Varimax rotated PCA solution was interpreted, which allowed a better explanation of the system since it considered the role of the latent factors with higher impact on the variation explanation and diminished the role of PCs with lower incidence.

All statistical analyses were carried out using STATISTICA 7.0 statistical software (StatSoft, Dell, Round Rock, TX, USA).

#### 4.2.3. Source Apportionment

The contribution of each, identified by principal components analysis factor, towards the general value of a certain parameter (variable, e.g., sum of total concentration) is an extremely important chemometric task. Usually, in pollution studies the identified factors (principal components) represent conditionally a possible pollution source. A well-defined factor structure makes it possible to create a specific receptor model for the assessment of the contribution of each factor to the total concentration, e.g., APCS (absolute principal components scores) model of Thurston and Spengler for airborne particulate matter [24]. First, a transformation of the factor scores towards zero is performed (target transformation). The absolute factor scores obtained are used as independent regressors in the regression models relating the total concentration with the contribution (usually in %) of each identified factor registered by PCA. This mode of receptor modeling (without knowing the preliminary composition of each source of pollution) ensures the determination of the element profile of a certain factor. The approach is also known as principal components regression (PCR).

## 5. Conclusions

The present study revealed some important relationships between the parameters used for the assessment of the soil quality around the biggest non-ferrous lead-zinc smelter in Bulgaria as well as between the sampling locations that represented the regions impacted by the smelter activity. A specific contribution of the study is the pollution source apportionment performed, which made it possible to gain specific information about the contribution of each identified source to the total concentration of a given pollutant.

Some significant results could be summarized as follows:

- By means of the source apportionment model and the average of the concentrations measured we have proven another source of copper impact different from the smelter itself; the copper concentration at the smelter sampling point is the lowest;
- Despite the fact that the smelter continues to be the major source of Zn, Cd, Hg and to lesser extent Pb pollution, comparison to previous data with these ones shows a significant decrease of the emissions of Zn, Cd and Pb;
- Though our results, do not relate the smelter production to copper pollution, it is readily seen that the copper content in the soil is drastically reduced. One of the reasons is the closure of the mine in the village of Zvezdel and the exchange of the ore source with another one. Another possible reason is that the copper source treated by us as geogenic and is related to the ore production and flotation around the mines near the settlements of Luki, Madan and Madjarovo;
- The higher levels of pollution at location AS (town of Assenovgrad) are due to the natural contribution of the underground waters and the Chaia River carrying waste from the lead-zink rich ores of Rhodopa Mountain where the mines and flotation facilities are located. The multivariate statistical expertise indicates this specificity by the contribution of the “natural source” in the apportionment models.

The chemometric expertise of the data set makes it possible to better interpret the specific relationships in a complex environmental system. Thus, it helps in solving problems and decision making for serious pollution problems.

## Figures and Tables

**Table 1:** The conditional groups and variables belonging to each of them.

Groups of Variables	Variables	Notation, e.g.,
soil structural (Hh, CEC, TEB) and physicochemical parameters	hydraulic activity; soil capacity; base exchange capacity; organic carbon; total organic carbon; pH, measured in KCl extract; pH, measured in water extract; saturation	Hh CEC TEB C_org TOC pH_KCl pH_w V
soil nutritional components	Mg, P, K, N	Mg_bio; N
soil pollution by heavy metals	Hg, Cd, Zn, Cu, Pb	Hg_soil
cabbage leaves pollution by heavy metals	Hg, Cd, Zn, Cu, Pb	Hg_leavs

**Table 2:** Factor loadings for 13 variables.

**Factor Loadings (Varimax normalized) (Marked loadings are >statistically significant)**					
**Variable**	**PC1**	**PC2**	**PC3**	**PC4**	**PC5**
pH_KCl	0.217	0.009	**0.919**	0.182	0.169
pH_w	0.171	0.118	**0.915**	0.122	0.257
C_org	**0.974**	0.016	0.119	−0.036	0.020
TOC	**0.974**	0.016	0.119	−0.036	0.020
N	**0.943**	−0.106	0.159	0.069	−0.065
P_bio	−0.055	0.275	0.107	**0.852**	0.036
K_bio	−0.308	−0.046	0.274	−0.081	**0.610**
Mg_bio	0.078	−0.291	0.480	**0.807**	−0.005
Zn_soil	−0.030	**0.876**	−0.040	0.118	0.357
Cd_soil	−0.190	**0.903**	0.043	−0.150	−0.282
Pb_soil	−0.069	0.442	−0.496	**0.593**	0.258
Cu_soil	0.173	−0.022	0.128	0.127	**0.893**
Hg_soil	0.202	**0.812**	0.085	0.416	−0.177
Expl. Var %	23.7	20.2	17.9	15.5	12.1

**Table 3:** Source apportionment models.

Variable	Unidentified Sources (Intercept)	Organic Source	Non-Ferrous Smelter Source	Acidic Source	Second Anthropogenic Contribution Source	Natural Source	R^2^
pH_KCl	4.7	8.6	-	78.6	4.3	3.8	0.84
pH_w	3.2	9.1	-	80.2	4.1	3.4	0.84
C_org	3.4	96.6	-	-	-	-	0.87
TOC	3.2	96.8	-	-	-	-	0.88
N	11.4	88.6	-	-	-	-	0.92
P_bio	8.6	-	15.9	-	75.5	-	0.82
K_bio	10.4	12.8	-	9.1	-	67.7	0.81
Mg_bio	7.3	-	-	10.6	82.1	-	0.82
Zn_soil	0.9	-	91.6	-	-	7.5	0.92
Cd_soil	5.4	-	82.9	-	5.6	6.1	0.88
Pb_soil	3.8	-	21.1	3.2	61.7	10.2	0.89
Cu_soil	9.3	-	-	3.3	3.1	84.3	0.93
Hg_soil	5.0	-	90.6	-	4.4	-	0.84

**Table 4:** Comparison of toxic metal pollution in the vicinity of the non-ferrous smelter for different time periods.

Locations	Metal Concentrations in the Topsoils (0–20) [mg kg^−1^]					Ref.
	Zn	Cd	Pb	Cu	Hg	
K *	103.74	0.17	17.66	33.55	0.033	present
*Kuklen*	158	3	97	49	-	[2]
KRM *	66.33	0.38	14.33	12.07	0.036	present
KAGRI *	384.60	2.22	19.69	8.00	0.106	present
*Kuklen around smelter*	5917	138	4892	666	-	[2]
AS *	409.70	0.97	31.68	25.22	0.072	present
*Boyntsi (14 km SE of Kuklen)*	340	5	225	71	*-*	[2]
PB *	330.40	0.81	14.73	30.76	0.043	present

* Average values of heavy metals concentrations in soils, collected for the period of three consecutive years for sampling locations of the present study are given.

## Supplementary Materials

The following are available online. Table S1 Input data set Table S2: Basic statistics, Figure S1a–e Factor loadings plots.
