# Supplementary material for: Chemometric Assessment of Soil Pollution and Pollution Source Apportionment for an Industrially Impacted Region around a Non-Ferrous Metal Smelter in Bulgaria
Source: Molecules. 2019 Mar 2;24(5):883. doi: 10.3390/molecules24050883 (PMC6429101; doi:10.3390/molecules24050883)
Supplement: Supplementary file 1 [file molecules-24-00883-s001.pdf]

molecules

Chemometric Assessment of Soil Pollution and Pollution Source Apportionment for an Industrially Impacted Region around a Non-Ferrous Metal Smelter in Bulgaria

Dimitar S. Dimitrov, Miroslava A. Nedyalkova , Borjana V. Donkova and Vasil D. Simeonov *

Faculty of Chemistry and Pharmacy, University of Sofia, 1 James Bourchier Blvd., Sofia 1164, Bulgaria; d_100@abv.bg *(D.S.D.)*; mici345@yahoo.com *(M.A.N.)*; bordonkova@abv.bg (B.V.D.); VSimeonov@chem.uni-sofia.bg *(V. D..S.)*

* Correspondence: VSimeonov@chem.uni-sofia.bg

Table S1. Input data set.

Variable	Valid N	Mean	Median	Minimum	Maximum	Variance	Std.Dev.
pH_KCl	16	6.0616	6.0525	3.89300	7.0900	0.52	0.7217
pH_w	16	6.3795	6.3500	4.25000	7.2200	0.45	0.6690
Hh	16	12.9100	7.9800	1.11000	49.5000	167.21	12.9310
TEB	16	32.1994	31.8100	22.45000	45.9000	26.90	5.1865
CEC	16	45.1094	42.4250	25.01000	82.3000	207.08	14.3904
V	16	75.4712	80.4319	39.85419	97.0789	261.76	16.1791
C_org	16	2.8987	1.7050	1.16000	9.6500	6.13	2.4750
TOC	16	4.9978	2.9397	2.00000	16.6379	18.21	4.2672
N	16	0.2835	0.1729	0.11290	1.2369	0.08	0.2788
P_bio	16	122.5524	112.2254	21.18310	240.2254	3197.38	56.5453
K_bio	16	126.5958	130.8186	17.88298	290.0532	5390.62	73.4208
Mg_bio	16	104.8944	105.0150	75.62000	135.4700	334.12	18.2790
Zn_leaves	16	50.6028	38.9061	15.96058	160.2024	1296.09	36.0012
Cd_leaves	16	0.1053	0.0603	0.00305	0.4462	0.02	0.1272
Pb_leaves	16	0.2941	0.1650	0.14000	1.4819	0.12	0.3488
Cu_leaves	16	2.1231	1.9993	0.32079	5.2578	2.07	1.4385

**Hg_leaves**	16	0.0035	0.0020	0.00200	0.0095	0.001	0.0028
**Zn_soil**	16	247.1426	122.3219	27.37953	883.1696	69325.03	263.2965
**Cd_soil**	16	0.8691	0.4818	0.10888	3.0467	0.78	0.8856
**Pb_soil**	16	18.9549	17.6218	6.41619	53.9246	121.23	11.0104
**Cu_soil**	16	21.5712	18.0087	0.79650	52.2290	251.66	15.8637
**Hg_soil**	16	0.0600	0.0495	0.02032	0.1459	0.00	0.0391

**Variable**	**Valid N**	**Mean**	**Median**	**Minimum**	**Maximum**	**Variance**	**Std.Dev.**
**pH_KCl**	16	6.06	6.05	3.89	7.09	0.52	0.72
**pH_w**	16	6.37	6.35	4.25	7.22	0.45	0.66
**Hh**	16	12.91	7.98	1.11	49.50	167.21	12.93
**TEB**	16	32.19	31.81	22.45	45.90	26.90	5.18
**CEC**	16	45.10	42.42	25.01	82.30	207.08	14.39
**V**	16	75.47	80.43	39.85	97.07	261.76	16.17
**C_org**	16	2.89	1.70	1.16	9.65	6.13	2.47
**TOC**	16	4.99	2.93	2.00	16.63	18.21	4.26
**N**	16	0.28	0.17	0.11	1.23	0.08	0.27
**P_bio**	16	122.55	112.22	21.18	240.22	3197.38	56.54
**K_bio**	16	126.59	130.81	17.88	290.05	5390.62	73.42
**Mg_bio**	16	104.89	105.01	75.62	135.47	334.12	18.27
**Zn_leaves**	16	50.60	38.90	15.96	160.20	1296.09	36.00
**Cd_leaves**	16	0.10	0.06	0.003	0.44	0.02	0.12
**Pb_leaves**	16	0.29	0.16	0.14	1.48	0.12	0.34
**Cu_leaves**	16	2.12	1.99	0.32	5.25	2.07	1.43
**Hg_leaves**	16	0.0035	0.002	0.002	0.009	0.001	0.0028
**Zn_soil**	16	247.14	122.32	27.37	883.16	69325.03	263.29
**Cd_soil**	16	0.86	0.48	0.10	3.04	0.78	0.88
**Pb_soil**	16	18.95	17.62	6.41	53.94	121.23	11.01
**Cu_soil**	16	21.57	18.01	0.79	52.22	251.66	15.86
**Hg_soil**	16	0.060	0.049	0.020	0.14	0.001	0.039

Table. S2. Basic statistics

Factor Loadings (Varimax normalized) (Marked loadings are >statistically significant)					
**Variable**	**PC 1**	**PC 2**	**PC 3**	**PC 4**	**PC 5**
pH_KCl	0.217	0.009	**0.919**	0.182	0.169
pH_w	0.171	0.118	**0.915**	0.122	0.257
C_org	**0.974**	0.016	0.119	-0.036	0.02
TOC	**0.974**	0.016	0.119	-0.036	0.02
N	**0.943**	-0.106	0.159	0.069	-0.065
P_bio	-0.055	0.275	0.107	**0.852**	0.036
K_bio	-0.308	-0.046	0.274	-0.081	**0.61**
Mg_bio	0.078	-0.291	0.48	**0.807**	-0.005
Zn_soil	-0.03	**0.876**	-0.04	0.118	0.357
Cd_soil	-0.19	**0.903**	0.043	-0.15	-0.282
Pb_soil	-0.069	0.442	-0.496	**0.593**	0.258
Cu_soil	0.173	-0.022	0.128	0.127	**0.893**
Hg_soil	0.202	**0.812**	0.085	0.416	-0.177
**Expl.Var %**	**23.7**	**20.2**	**17.9**	**15.5**	**12.1**

Figure S1. Factor loadings plots.

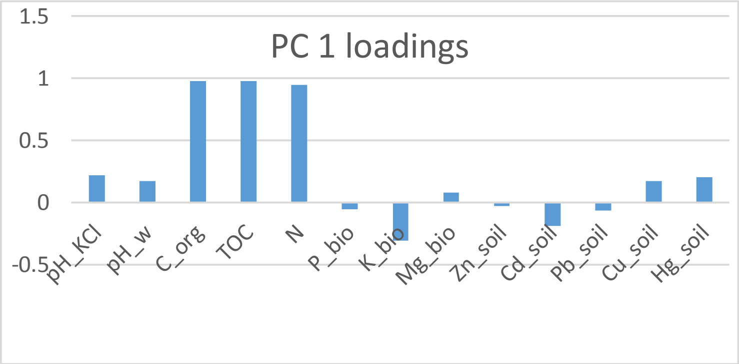

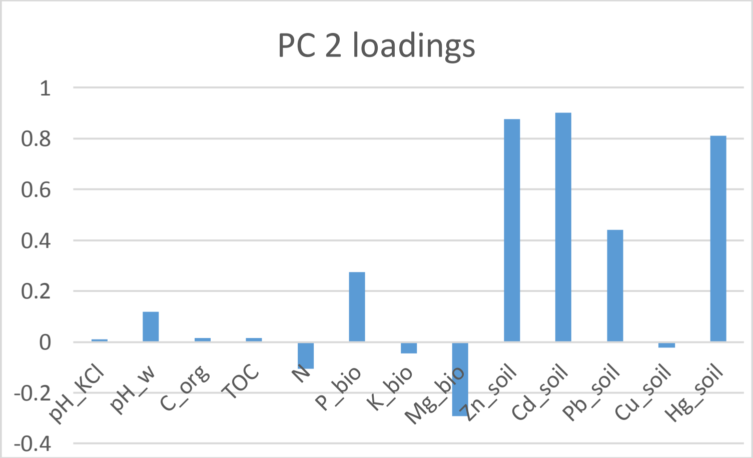

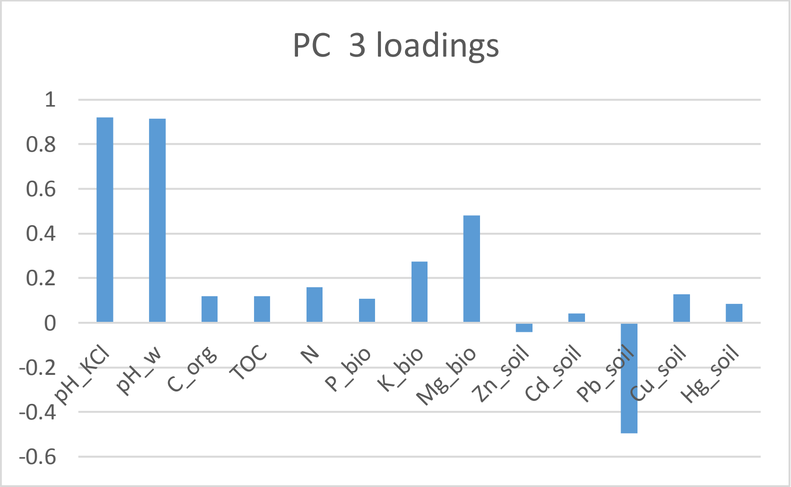
PC 3 loadings
1
0.8
0.6
0.4
0.2
0
-0.2
-0.4
-0.6
pH_KCl
pH_w
C_org
TOC
N
P_bio
K_bio
Mg_bio
Zn_soil
Cd_soil
Pb_soil
Cu_soil
Hg_soil

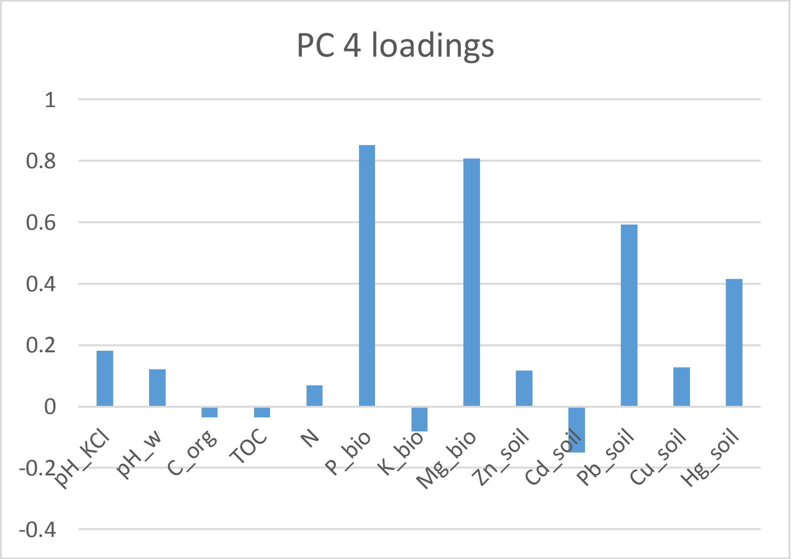
PC 4 loadings
1
0.8
0.6
0.4
0.2
0
-0.2
-0.4
pH_KCl
pH_w
C_org
TOC
N
P_bio
K_bio
Mg_bio
Zn_soil
Cd_soil
Pb_soil
Cu_soil
Hg_soil

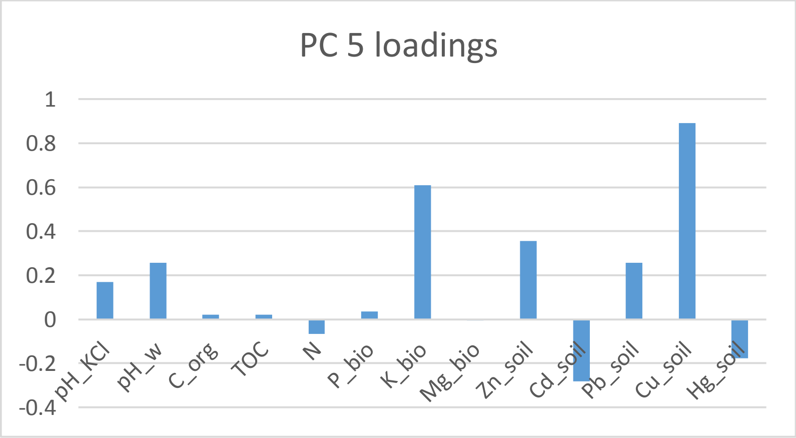
PC 5 loadings
1
0.8
0.6
0.4
0.2
0
-0.2
-0.4
pH_KCl
pH_w
C_org
TOC
N
P_bio
K_bio
Mg_bio
Zn_soil
Cd_soil
Pb_soil
Cu_soil
Hg_soil
